# Sex influences DNA methylation and gene expression in human skeletal muscle myoblasts and myotubes

**DOI:** 10.1186/s13287-018-1118-4

**Published:** 2019-01-15

**Authors:** Cajsa Davegårdh, Elin Hall Wedin, Christa Broholm, Tora Ida Henriksen, Maria Pedersen, Bente Klarlund Pedersen, Camilla Scheele, Charlotte Ling

**Affiliations:** 1 Department of Clinical Sciences, Lund University Diabetes Centre, Lund University, Scania University Hospital, 20502 Malmö, Sweden; 2 Department of Endocrinology, Rigshospitalet, 2100 Copenhagen, Denmark; 3 The Centre of Inflammation and Metabolism and the Centre for Physical Activity Research, Rigshospitalet, University of Copenhagen, Copenhagen, Denmark; 4 Novo Nordisk Foundation Center, Section for Basic Metabolic Research, Faculty of Health and Medical Science, University of Copenhagen, Copenhagen, Denmark

**Keywords:** Gender, Muscle stem cells, Myoblasts, Myotubes, Skeletal muscle, Epigenetics, mRNA expression, Transcriptome, Epigenome, Myogenesis

## Abstract

**Background:**

Sex differences are known to impact muscle phenotypes, metabolism, and disease risk. Skeletal muscle stem cells (satellite cells) are important for muscle repair and to maintain functional skeletal muscle. Here we studied, for the first time, effects of sex on DNA methylation and gene expression in primary human myoblasts (activated satellite cells) before and after differentiation into myotubes.

**Method:**

We used an array-based approach to analyse genome-wide DNA methylation and gene expression in myoblasts and myotubes from 13 women and 13 men. The results were followed up with a reporter gene assay.

**Results:**

Genome-wide DNA methylation and gene expression differences between the sexes were detected in both myoblasts and myotubes, on the autosomes as well as the X-chromosome, despite lack of exposure to sex hormones and other factors that differ between sexes. Pathway analysis revealed higher expression of oxidative phosphorylation and other metabolic pathways in myoblasts from women compared to men. Oxidative phosphorylation was also enriched among genes with higher expression in myotubes from women. Forty genes in myoblasts and 9 in myotubes had differences in both DNA methylation and gene expression between the sexes, including *LAMP2* and *SIRT1* in myoblasts and *KDM6A* in myotubes. Furthermore, increased DNA methylation of *LAMP2* promoter had negative effects on reporter gene expression. Five genes (*CREB5*, *RPS4X, SYAP1*, *XIST*, and *ZRSR2)* showed differential DNA methylation and gene expression between the sexes in both myoblasts and myotubes. Interestingly, differences in DNA methylation and expression between women and men were also found during differentiation (myoblasts versus myotubes), e.g., in genes involved in energy metabolism. Interestingly, more DNA methylation changes occur in women compared to men on autosomes.

**Conclusion:**

All together, we show that epigenetic and transcriptional differences exist in human myoblasts and myotubes as well as during differentiation between women and men. We believe that these intrinsic differences might contribute to sex dependent differences in muscular phenotypes.

**Electronic supplementary material:**

The online version of this article (10.1186/s13287-018-1118-4) contains supplementary material, which is available to authorized users.

## Background

Sex contributes to differences in many aspects of metabolism and diseases such as obesity and diabetes [1], and it contributes to differences in skeletal muscle morphology and metabolism [2, 3]. Additionally, age-related muscle decline and remodelling differ in women versus men [4].

**Fig. 1:**
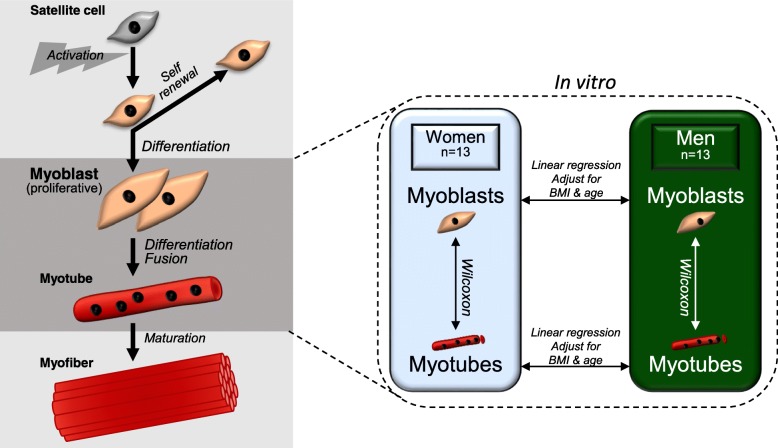
Schematic figure of myogenesis as well as the study design. The leftmost part of the figure shows the adult myogenic process, from activation of skeletal muscle stem cells to a mature myofibre, as it occurs naturally in vivo. The rightmost part shows our study design covering the different analyses of genome-wide DNA methylation and gene expression in primary human myoblasts and in vitro differentiated myotubes from women and men. BMI, body mass index; Wilcoxon, Wilcoxon signed-rank test

Adult skeletal muscle stem cells, so-called satellite cells, are responsible for regeneration and maintenance of skeletal muscle [5], thereby contributing to a healthy muscle phenotype. The satellite cells are activated in response to stress, after, e.g. injury or exercise, which initiates proliferation. Asymmetric cell division gives rise to new stem cells as well as muscle progenitors, called myoblasts, that eventually withdraw from the cell cycle, differentiate, fuse into myotubes and later mature into myofibres (Fig. 1) [6].

Studies in mice have identified sex differences in skeletal muscle regeneration. Female myoblasts transplanted with higher efficiency regardless sex of the host, and transcriptional sex differences in the myoblasts were detected [7]. However, there is limited knowledge about the impact of sex on human myogenesis. Nevertheless, sex-specific differences in human skeletal muscle tissue transcriptome have been shown at baseline as well as in response to exercise and with age [8, 9]. Sex-specific muscle phenotypes may arise due to, e.g. hormonal effects of androgens or oestrogen, metabolic programming, or genetic differences [1]. Furthermore, sex-biased differentially expressed genes in skeletal muscle are located both on the autosomal chromosomes and the X-chromosome [8, 9].

Epigenetic modifications, such as DNA methylation, are important for inactivation of one X-chromosome in women, regulation of gene expression and differentiation of stem cells [10]. DNA methylations can be influenced by the environment and change with age [11]. We have previously shown genome-wide DNA methylation changes during differentiation of primary human myoblasts into myotubes, and obesity-associated epigenetic programming of these cells [12]. Sex-specific DNA methylation has been observed in other tissues, such as pancreatic islets, blood and liver among others [13–17]. However, it has not yet, to our knowledge, been studied in human muscle stem cells.

We hypothesise that sex influences DNA methylation and gene expression in human skeletal muscle stem cells and thereby affects myogenesis and muscle phenotypes. Our aim is to compare the genome-wide DNA methylome in primary human myoblasts and in vitro differentiated myotubes from women versus men, and relate epigenetic differences to gene transcription. We also studied the direct effect of increased methylation on transcriptional activity in cultured myoblasts.

## Methods

### Study subjects’ characteristics

Women and men were selected from a study population which has been previously described in detail elsewhere [12, 18, 19]. Thirteen women and 13 men without any known disease (except 6 individuals of each sex with obesity) were selected based on matching body mass index (BMI) and age from a subset of the cohort where DNA methylation and gene expression array data already existed (part of these data have been published previously [12] (Table 1). Data were not available for one man for the following phenotypes: glucose 2 h, Insulin 0 h, Insulin 2 h and homeostasis model assessment of insulin resistance and β-cell function (HOMA-IR and HOMA-B). Furthermore, mRNA expression data were not available for two women.

**Table 1:** Phenotypes of individuals included in the study

Phenotype	Women (*n* = 13)	Men (*n* = 13)	*p* value
Age (years)	53.5 ± 7.9	52.4 ± 5.3	0.69
BMI (kg/m^2^)	30.1 ± 6.8	28.8 ± 5.1	0.59
Weight (kg)	84.9 ± 24.3	96.9 ± 17.2	0.16
Waist-hip ratio (waist/hip)	0.85 ± 0.1	0.96 ± 0.07	5.1 × 10^− 03^
Hip circumference (cm)	111.9 ± 14.0	107.5 ± 8.4	0.34
Waist circumference (cm)	96.5 ± 22.1	103.5 ± 13.4	0.34
Android fat mass (kg)	3.2 ± 2.1	3.1 ± 1.6	0.94
Gynoid fat mass (kg)	6.8 ± 2.3	4.1 ± 1.4	1.8 × 10^− 03^
Whole body fat mass (kg)	35.3 ± 16.0	26.5 ± 10.8	0.12
Whole body fat-free mass (kg)	45.8 ± 8.7	66.5 ± 7.5	1.0 × 10^− 06^
Glucose 0 h (mmol/l)	4.7 ± 0.5	5.1 ± 0.5	0.06
Glucose 2 h (mmol/l) *	5.5 ± 1.5	5.3 ± 1.3	0.75
Insulin 0 h (pmol/l) *	48.8 ± 33.6	66.3 ± 36.1	0.22
Insulin 2 h (pmol/l) *	311.7 ± 176.8	373.4 ± 326.1	0.56
HOMA-IR*	1.5 ± 1.1	2.2 ± 1.3	0.17
HOMA-β*	111.1 ± 60.8	132.2 ± 92.3	0.50
P-cholesterol (total mmol/l)	5.2 ± 0.8	5.3 ± 1.0	0.90
P-cholesterol HDL (mmol/l)	1.7 ± 0.6	1.4 ± 0.4	0.11
P-cholesterol LDL (mmol/l)	3.0 ± 0.4	3.4 ± 0.9	0.17
Systolic blood pressure (mmHg)	127.4 ± 12.7	144.5 ± 12.8	2.2 × 10^− 03^
Diastolic blood pressure (mmHg)	86.5 ± 10.7	90.6 ± 8.1	0.28
Heart rate (beats/min)	68.5 ± 8.8	57.3 ± 10.9	8.5 × 10^− 03^
VO_2_ max (l/min)	2.1 ± 0.7	2.9 ± 0.8	0.01
VO_2_ max per kg (ml/min/kg)	24.7 ± 8.7	30.5 ± 11.4	0.15

Data are presented as mean ± SD. *p* values were calculated using *t*-tests

*Data from 12 men are available for these characteristics

### Human muscle stem cell isolation and culture

A detailed description of the method can be found in previous publications [12, 18]. In short, satellite cells were isolated from vastus lateralis. Myoblasts were seeded on BD Matrigel™ (BD Biosciences, NJ, USA), cultured in HAM/F10 supplied with 20% fetal bovine serum (FBS) and 1% penicillin/streptomycin (PS) and harvested when less than 50% confluent. At 70–80% confluency, growth medium was switched to DMEM (1.0 g/L glucose) supplied with 10% FBS and 1% PS until cells were confluent and aligned. Differentiation was then enhanced by switching to DMEM (4.5 g/L glucose) supplied with 2% horse serum and 1% PS for 5 days for myotube formation. Full differentiation was determined via visual confirmation where approximately 70% of the myotubes should have > 3 nuclei. DNA and RNA was extracted from the cells using the DNeasy blood and tissue kit (Qiagen, Hilding, Germany) and Trizol in combination with RNeasy minElute Cleanup kit (Qiagen), respectively.

Purity of the cultures in this cohort has been assessed by flow cytometry and published elsewhere [12]. All cultures expressed the myogenic marker CD56, while the endothelial and haematopoietic markers CD31 and CD45 were not detected.

### DNA methylation analysis

DNA methylation was analysed genome-wide using the Infinium HumanMethylation450K BeadChip (Illumina, Inc., CA, USA). The method has been described in detail elsewhere [12]. Myoblasts and myotubes from the same individual were analysed on the same chip. Women and men were equally distributed on the different chips. Y-chromosome annotated probes were removed from initial analysis, together with rs-probes, non-CpG probes and cross-reactive probes with at least 49-base pair (bp) match [20]. Mean detection *p* value cut off was set to 0.01. Beta-mixture quantile normalisation (BMIQ) [21] was used to normalise between Infinium I and Infinium II assays. COMBAT [22] was used to correct for batch in non-paired analysis, i.e. women versus men. Methylation for each analysed site is presented as β value ranging from 0 to 1 (0–100% methylation). In total 458,475 probes were analysed for all chromosomes, 447,867 for autosomes and 10,608 for the X-chromosome. Y-chromosome DNA methylation data were normalised (including all chromosomes) and analysed separately.

The Illumina 450k array annotates the probes to different genomic regions [23]; in relation to the transcription start sites (TSS) either at 0 to 200 bp (TSS200) or between 200 and 1500 bp upstream of the TSS (TSS1500), to 5′ untranslated region (5′UTR), 1st exon, gene body (introns and exons, except 1st exon), 3′UTR or intergenic (not annotated to any gene/gene region). The probes are also annotated to CpG island regions; inside the CpG island itself, defined as 500 bp with GC content higher than 50% and observed/expected CpG ratio > 0.6 [23]. The 2-kb regions directly flanking the CpG island, either upstream (northern (N)) or downstream (southern (S)) of the islets, are called shores and the 2-kb regions flanking the shores are called the northern and southern shelf.

### mRNA expression analysis

mRNA expression was analysed genome-wide using HumanHT-12 Expression BeadChip (Illumina). The array contains 47,231 probes and those with mean detection *p* value > 0.01 for more than 60% of the samples were filtered out. Data were background corrected, log2 transformed and quantile normalised using lumi package [24] in R (Additional file 1). In total, 16,955 transcripts, corresponding to 10,925 unique genes, were found expressed after quality control (QC). Of these genes, 9766 overlapped with genes from the methylation array.

### Gene set enrichment analysis (GSEA)

GSEA was analysed using GSEA software [25, 26] to find enriched KEGG pathways. This software enabled us to analyse the complete expression data set using a pre-ranked list, based on t-statistics, where duplicate gene symbols were removed based on *p* value (lowest *p* value was kept in the data set). Default setting was used, except for minimal size of gene sets, which was set to two genes.

### Luciferase assay

The luciferase assay method to measure the direct DNA methylation effect on gene expression has been described in detail elsewhere [12, 13]. In short, 1500-bp fragments of human promoters from *LAMP2* and *RPS4X* were cloned into a CpG-free luciferase vector (pCpGL-basic) [27] and amplified by GenScript (GenScript USA Inc., Piscataway, NJ, USA). The constructs where then either mock methylated or methylated in vitro using the methyltransferases SssI, HhaI or HpaII (2.5 U/μg DNA) (New England Biolabs), which methylate cytosines in the following context: CG, GCGC and CCGG, respectively. Mouse myoblasts (C2C12) cultured in DMEM (4.5 g/L glucose) supplied with 10% FBS and 1% PS in a 96-well plate were co-transfected with either 150 ng DNA/well (*LAMP2*) or 50 ng DNA/well (*RPS4X*) of the pCpGL vector constructs together with a Renilla luciferase control reporter vector (Promega, Madison, WI, USA) using FuGeneHD (Promega). Luciferase and Renilla activity were measured 48 h post transfection in cell lysate using Dual-Luciferase Reporter Assay System (Promega). Luciferase activity was calculated as the ratio between the reporter gene firefly luciferase and the control vector Renilla luciferase.

### DNA methylation and mRNA expression data in skeletal muscle

DNA methylation and gene expression data from non-diabetic individuals in a previously published study were used to study differences between sexes in skeletal muscle [28]. DNA methylation data were analysed by Infinium HumanMethylation450K BeadChip (Illumina) and gene expression data using GeneChip Human Gene 1.0 ST array by Applied Biosystems (Foster City, CA, USA). DNA methylation data for myotubes and skeletal muscle were merged based on Illumina TargetID and the expression data based on gene symbol.

### Statistical analysis

*t*-tests were used to analyse differences in phenotypes between men and women and the data are presented as mean ± standard deviation (SD).

For DNA methylation and gene expression analyses, *p* values were calculated with linear regression corrected for age and BMI in the comparisons between women and men, and with a paired non-parametric test (Wilcoxon) for comparisons between myoblasts and myotubes from the same individuals. Frequencies of significant CpG sites in different genomic regions and regions in relation to CpG island were analysed with chi-square tests against the expected frequency (5% for *p* < 0.05) of all analysed sites. A false discovery rate (FDR) (Benjamini–Hochberg procedure) was performed to correct for multiple testing and to reduce the amount of false positives in all the array data (autosomal chromosomes and X-chromosome together) and GSEA. Separate FDR analyses were run for the Y-chromosome in men. FDR analyses of muscle data were performed after filtering on CpG sites/genes that overlapped with significant results in myotubes. Principal component (PC) analyses were performed after batch correction and the top PCs correlated with sex. Correlations between expression data from all samples were calculated using Pearson correlation analyses.

Luciferase assay results were analysed using paired *t*-test against the control.

## Results

### Differences in clinical phenotypes between women and men

Human myoblasts and myotubes derived from human satellite cells from 13 healthy women and 13 healthy men were included in the study (Fig. 1). Their characteristics are shown in Table 1. There were no differences in age or BMI between the groups. Women had higher gynoid fat mass and heart rate (HR), while men were taller and had higher whole-body fat-free mass, systolic blood pressure, waist-hip ratio and VO_2_ max (Table 1).

### Sex-specific differences in DNA methylation and gene expression in human myoblasts

We started to analyse DNA methylation in cells harvested as proliferating myoblasts using Illumina 450k array (Fig. 1). Methylation data for a total number of 458,475 CpG sites, 447,867 on autosomal chromosomes and 10,608 on the X-chromosome were obtained from all individuals.

To examine potential sources of variation, we performed PC analysis of the methylation data set in myoblasts and correlated the top PCs with sex. The samples clustered based on sex (Additional file 2), as expected when methylation of the X-chromosome is included [13, 17]. Furthermore, sex was significantly correlated with the third PC (*p* = 1.47 × 10^− 5^), suggesting that sex has a relatively large impact on DNA methylation in myoblasts.

**Fig. 2:**
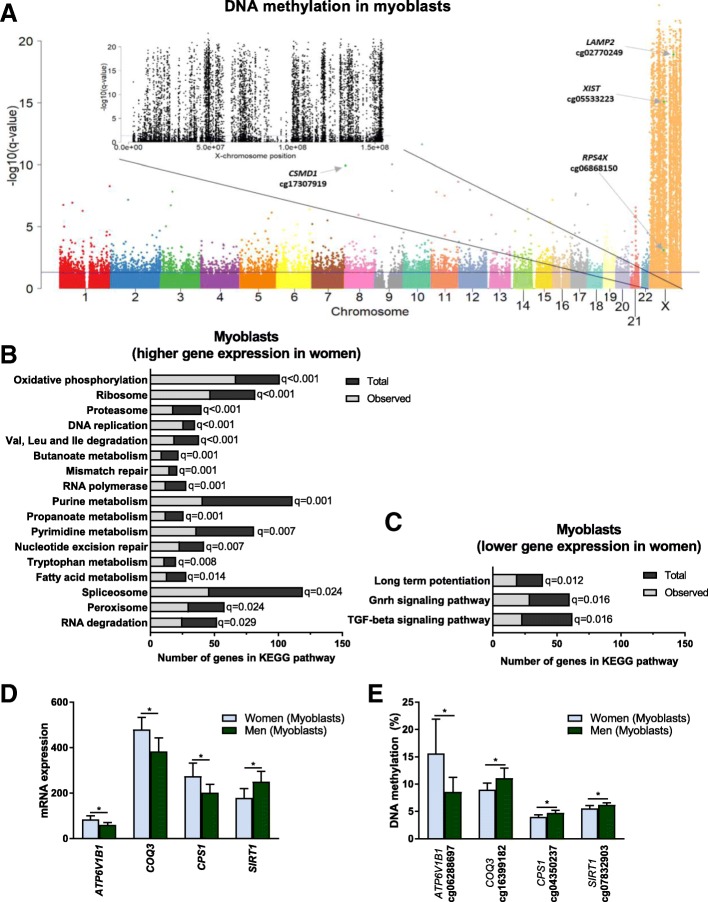
Differential DNA methylation and gene expression in human myoblasts from women and men. **a** Manhattan plot of all analysed CpG sites for DNA methylation in myoblasts from women versus men. The blue line indicates the threshold for significance (*q <* 0.05). Green dots indicated with arrows are selected CpG sites for some genes of interest. Manhattan plot with black points are CpG sites only on the X-chromosome. *N* = 13 for both women and men. **b**, **c** A selection of relevant significant KEGG pathways (*q <* 0.05) from GSEA of expression data in myoblasts from women versus men (*N* = 11–13). Total, total number of genes in the pathway in the analysed dataset; Observed, observed number of genes in the dataset contributing to the enrichment; Val, valine; Leu, leucine; Ile, isoleucine; Gnrh, gonadotropin-releasing hormone; TGF-beta, transforming growth factor-beta. **d** Gene expression of selected genes in metabolic pathways and **e** DNA methylation of significant CpG sites annotated to these genes in myoblasts from women and men. *, *q <* 0.05, *N* = 11–13. Data are presented as mean ± SD

We continued to analyse the impact of sex on methylation of individual CpG sites in the myoblasts using linear regression. Based on FDR of less than 5% (*q* < 0.05), 12,177 CpG sites had significant differences, in methylation between women and men (Fig. 2a and Additional file 3). Of these, 5762 CpG sites were located on the autosomal chromosomes (*q* < 0.05) with absolute differences in methylation up to 46.1%. The majority (62%) had higher methylation in women compared to men. On the X-chromosome, 6415 CpG sites had different methylation (q < 0.05) between women and men with absolute differences up to 60.2%. Here, 78% had a higher methylation level in women, and notably 22% had a higher methylation level in men. More differences on the X-chromosome compared to autosomal chromosomes are expected due to the X-chromosome inactivation in women [13, 17, 29].

The CpG sites on the array have been annotated to different functional genomic regions as well as regions based on CpG content [23], see Additional file 4 for details. DNA methylation in these different regions might have diverse effects on transcription of their corresponding genes [10]. Hence, we evaluated where CpG sites with significant differences (*q* < 0.05) in DNA methylation between women and men are located. In general, we found more CpG sites than expected by chance close to TSS and in CpG islands on both autosomal chromosomes and the X-chromosome (Additional file 4).

To investigate if sex also affects gene expression in myoblasts, we used a microarray approach with mRNA from the same samples as for DNA methylation. Correlations of data from all samples were high (Additional file 1) and a PC analysis revealed significant correlation between sex and the third PC (*p* = 0.01) as well as the fourth PC (*p* = 8.64 × 10^− 4^).

We then performed GSEA [26] to analyse enrichment of KEGG pathways in the whole expression dataset. Twenty pathways were enriched (*q <* 0.05) with higher expression levels in women compared to men, including several pathways related to the cell cycle as well as metabolism of energy, proteins and fatty acids (Fig. 2b and Additional file 5). In contrast, only four pathways were enriched with higher expression in men compared to women (*q <* 0.05), mostly related to cell-cell communication, e.g. transforming growth factor-beta (TGF-beta) signalling (Fig. 2c and Additional file 5).

Next, we analysed expression of individual genes in myoblasts and found 137 unique genes with significantly different expression (*q <* 0.05) between women and men (Additional file 6). Importantly, 124 of these genes were located on autosomal chromosomes and include genes known to be involved in myogenesis and energy metabolism, in line with GSEA. For example, *CTF1* had higher expression in men and *MAMSTR*, *TFB1M* and *LDHB* had higher expression in women.

**Fig. 3:**
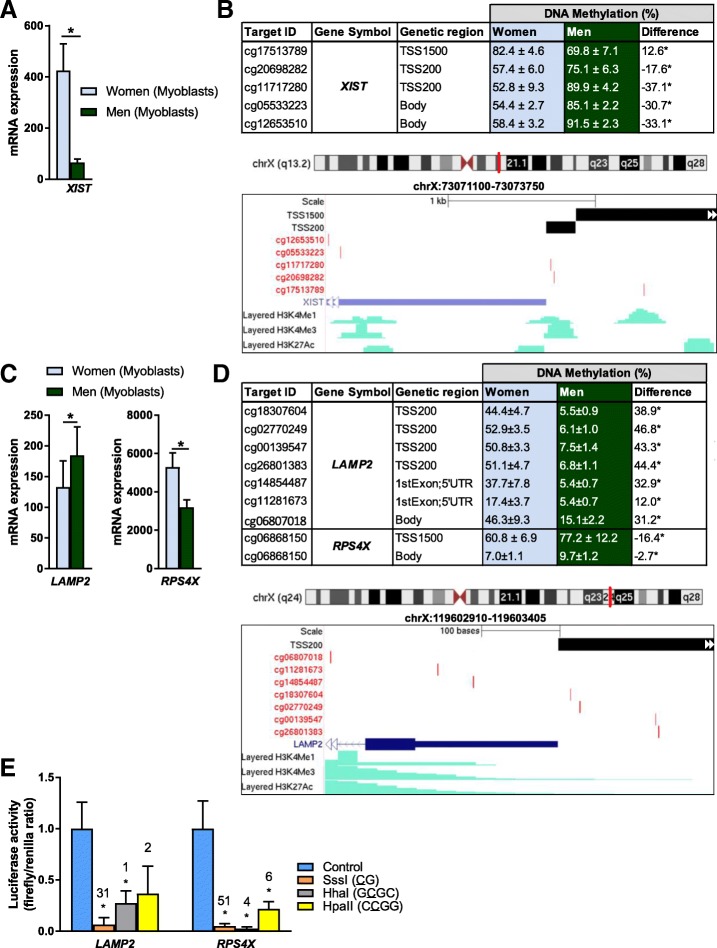
Genes with both differential DNA methylation and gene expression in human myoblasts from women versus men. **a**–**d** Bar graphs showing mRNA expression and tables showing DNA methylation of **a**, **b** *XIST* and **c**, **d** *LAMP2* and *RPS4X* in myoblasts from women versus men. Only data for CpG sites with significant differences between women and men are included. Below the tables are UCSC genome browser tracks for **b** XIST and **d** *LAMP2* showing the location of the promoter region, CpG sites in the table above, the Refseq gene and histone modifications from ENCODE data in skeletal muscle myoblasts (HSMM). Red line on the chromosome above indicates location on the X-chromosome. *N* = 11–13, *q < 0.05; H3K4me1, Histone 3 lysine 4 (H3K4) methylation; H3K4me3, H3K4 trimethylation; H3K27ac, H3K27 acetylation; TSS200/TSS1500, 0–200 bp and 200–1500 bp upstream of the transcription start site (TSS), respectively. **e** In vitro methylation of *LAMP2* and *RPS4X* promoters reduced expression (activity) of a luciferase reporter gene. Numbers above the bars indicate the number of cytosines targeted by each enzyme in the promoter sequences (SssI: all CG, HhaI: GCGC and HpaII: CCGG). *p < 0.05, *N* = 5–6. Mean of the control for each gene was set to 1. Data are presented as mean ± SD

DNA methylation is known to affect gene expression [10], and we therefore combined our gene expression data with the DNA methylation data. Forty unique genes had significant changes (*q <* 0.05) in both mRNA expression and methylation (Additional file 7), whereas 10 were located on the X-chromosome. Also here, we found several genes involved in metabolic pathways. For example, *SIRT1* had higher expression in men, while *ATP6V1B1*, *CPS1* and *COQ3* had higher expression in women (Fig. 2d, e). Genes on the X-chromosome with both differential gene expression and DNA methylation included, e.g. *XIST*, involved in X-chromosome inactivation [30], *LAMP2* and *RPS4X* (Fig. 3a–d).

### DNA methylation of *LAMP2* and *RPS4X* promoter regions directly affects transcriptional activity in myoblasts

DNA methylation in promoter regions is associated with low gene expression [10]. In order to study direct effects of promoter DNA methylation on gene expression level in myoblasts, we designed a luciferase assay by cloning the *LAMP2* and *RPS4X* promoter regions, respectively, into a CpG-free vector. *LAMP2* encodes lysosomal associated membrane protein 2 (LAMP-2) involved in autophagy, a process important for proper myogenesis [31–33]. *LAMP2* had a higher expression level, accompanied with lower methylation level in the proximal promoter (TSS200), in myoblasts from men (Fig. 3c, d). *RPS4X* encodes a protein of the 40S ribosomal subunit and is known to escape X-chromosome inactivation [34]. As seen in Fig. 3c, d, *RPS4X* exhibited higher expression and lower promoter methylation in women. The luciferase assay clearly showed that increased methylation in the promoter region of these genes reduced expression of the reporter gene (Fig. 3e). This result supports that methylation in these promoters directly regulates the expression of their genes.

### Sex-specific differences in DNA methylation and gene expression in human myotubes

We continued to analyse DNA methylation in myotubes derived from myoblasts of the 13 women and 13 men (Fig. 1). The samples clustered based on sex, as was also seen for the myoblasts, (Additional file 2), and sex was again significantly correlated with the third PC (*p* = 1.47 × 10–5).

**Fig. 4:**
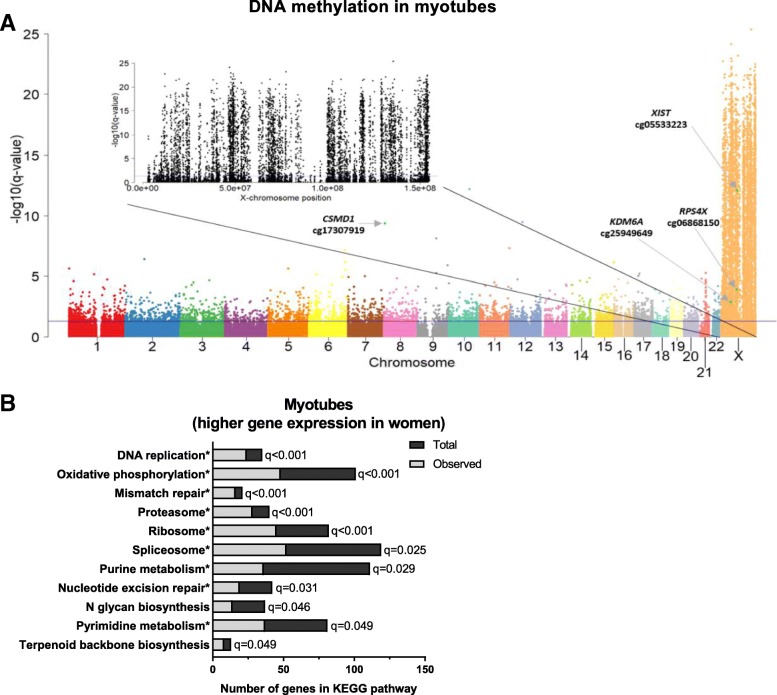
Differential DNA methylation and gene expression in human myotubes from women and men. **a** Manhattan plot of all analysed CpG sites for DNA methylation between women and men in myotubes. The blue line indicates the threshold for significance (*q <* 0.05). Green dots indicated with arrows are selected CpG sites for some genes of interest. Manhattan plot with black points are CpG sites only on the X-chromosome. *N* = 13 for both women and men. **b** A selection of relevant significant KEGG-pathways (*q <* 0.05) from GSEA of expression data for women versus men in myotubes (*N* = 11–13). *, pathways enriched in both myoblasts and myotubes; Total, total number of genes in the pathway in the analysed dataset; Observed, observed number of genes in the dataset contributing to the enrichment

Between women and men, 11,097 CpG sites had significantly different DNA methylation levels (*q <* 0.05) (Fig. 4a and Additional file 3). Of these CpG sites, 4918 were located on the autosomal chromosomes with absolute differences in methylation up to 39.2%, and 6179 sites were located on the X-chromosome, with absolute methylation differences up to 58.9%. Similar to the myoblasts, many significant sites on the X-chromosome (19%) had higher methylation level in men. We also found a similar pattern between myoblasts and myotubes when we analysed the distribution of these significant sites on the X-chromosome in relation to gene regions and CpG island regions (Additional file 4). However, these consistent patterns were not seen for the autosomal chromosomes (Additional file 4), indicating that sex-specific methylation differences on the X-chromosome are more persistent than on autosomal chromosomes during differentiation.

We proceeded to study the impact of sex on gene expression in myotubes. Correlations of data from all samples were high (Additional file 1), and sex correlated significantly with PC 3 to 5 (*p* = 0.003, 0.005 and 0.03) in a PC analysis.

GSEA of gene expression data in myotubes revealed 15 significant KEGG pathways (*q <* 0.05) that were upregulated in women compared to men (Fig. 4b and Additional file 5). Of note, 12 of these were also upregulated in myoblasts, including oxidative phosphorylation and DNA replication. However, no gene set was downregulated in myotubes from women compared to men.

Twenty-two unique genes were differently expressed (*q <* 0.05) between women and men in myotubes (Additional file 6). Of these, 13 genes were located on autosomal chromosomes and 9 genes on the X-chromosome.

Further, 9 unique genes in the myotubes had significant changes (*q <* 0.05) in both expression and DNA methylation between women and men (Additional file 7). Seven of these genes were located on the X-chromosome, including *KDM6A* (Fig. 4a) which is a histone demethylase important for myogenesis [35] and previously shown to be differentially expressed and methylated between women and men in other tissues [13, 17].

### Sex differences in DNA methylation and gene expression in human myoblasts remain and arise in myotubes

**Fig. 5:**
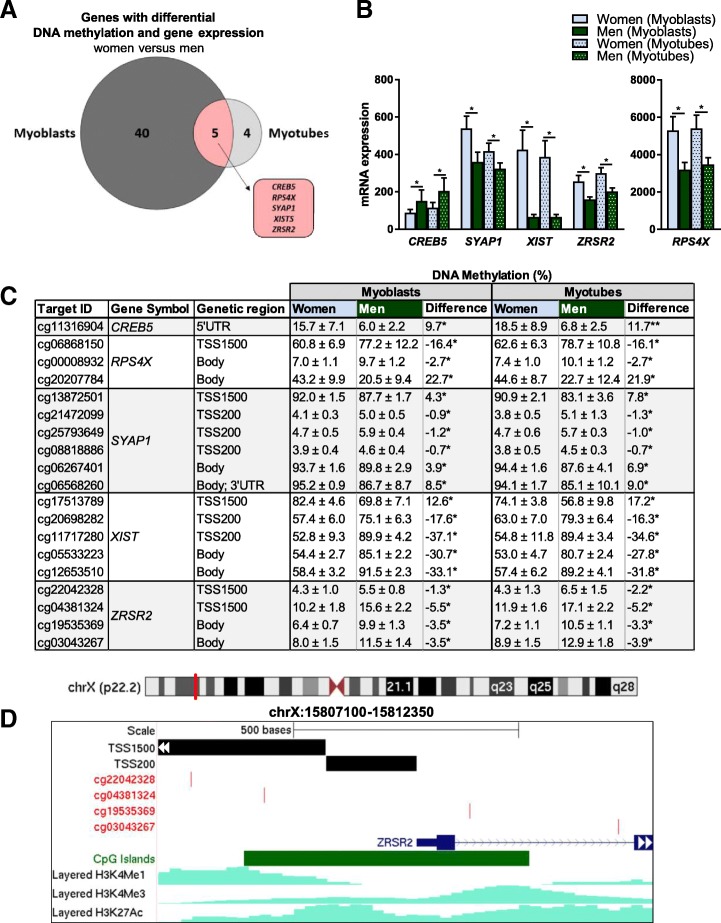
Differences in expression and methylation between women and men in both myoblasts and myotubes. **a** Overlap of number of unique genes with significant differences (*q <* 0.05) in both gene expression and DNA methylation in myoblasts and myotubes from women versus men. **b** Gene expression and **c** DNA methylation in myoblasts and myotubes of *CREB5*, *SYAP1*, *XIST*, *ZRSR2* and *RPS4X*. Only CpG sites with significant differences in DNA methylation in both myoblasts and myotubes are shown. **d** Genome browser track for *ZRSR2* showing the location of the promoter region, CpG sites in the table above, the Refseq gene and histone modifications from ENCODE data in skeletal muscle myoblasts (HSMM). Red line on the chromosome above indicates location on the X-chromosome. *N* = 11–13, *q < 0.05; H3K4me1, Histone 3 lysine 4 (H3K4) methylation; H3K4me3, H3K4 trimethylation; H3K27ac, H3K27 acetylation; TSS200/TSS1500, 0–200 bp and 200–1500 bp upstream of the transcription start site (TSS), respectively. Data are presented as mean ± SD

To better understand which of the sex differences seen in myoblasts that persist after differentiation into myotubes, we compared our significant (*q <* 0.05) DNA methylation and gene expression hits in myoblasts and myotubes (Fig. 5a). Interestingly, more significant methylation and expression differences were seen in myoblasts compared to myotubes, both on autosomal chromosomes and on the X-chromosome. While 25% (1453) of the CpG sites that were differentially methylated in myoblasts on autosomal chromosomes remained significant in myotubes, 92% (5912 CpG sites) overlapped on the X-chromosome. Again, this suggests that sex differences in DNA methylation on the X-chromosome, compared to the autosomal chromosomes, are more persistent during myogenesis.

Nine genes were differentially expressed (*q <* 0.05) between women and men in both myoblast and myotubes, and 5 of them (*CREB5*, *SYAP1*, *XIST*, *ZRSR2* and *RPS4X*) also showed differences in DNA methylation (*q <* 0.05) (Fig. 5a–d). All of these genes, except *CREB5*, are located on the X-chromosome. They are involved in, e.g. splicing, X-chromosome inactivation and transcriptional activation [30, 36–38], suggesting that they may further regulate expression of other genes. Together, the data show that many sex differences in DNA methylation and gene expression, both on the X-chromosome and autosomal chromosomes, in human myoblasts remain after differentiation into myotubes. The findings also indicate that sex differences arise during cell specification, since several differences between women and men in myotubes were not seen in the myoblasts.

### Sex-specific differences in global DNA methylation in human myoblasts and myotubes

Previous studies have shown sex differences in DNA methylation of specific functional genomic elements and CpG island regions [13, 17]. We therefore analysed the average DNA methylation levels in the myoblasts and myotubes from women and men for each of these regions (Additional file 8). Interestingly, men had higher methylation than women in 3′ untranslated regions (UTR) and shelves on the X-chromosome. Women had higher methylation in the other regions, as expected due to the role of DNA methylation for X-chromosome inactivation [13, 17, 29]. On the autosomal chromosomes, methylation levels in myotubes, but not myoblasts, were close to significantly higher in women compared to men in gene bodies, 3′ UTR, intergenic regions and CpG islands (*q* = 0.051). These data support that global methylation differences may arise during muscle differentiation.

### Changes in DNA methylation and gene expression during myogenesis in women and men

**Fig. 6:**
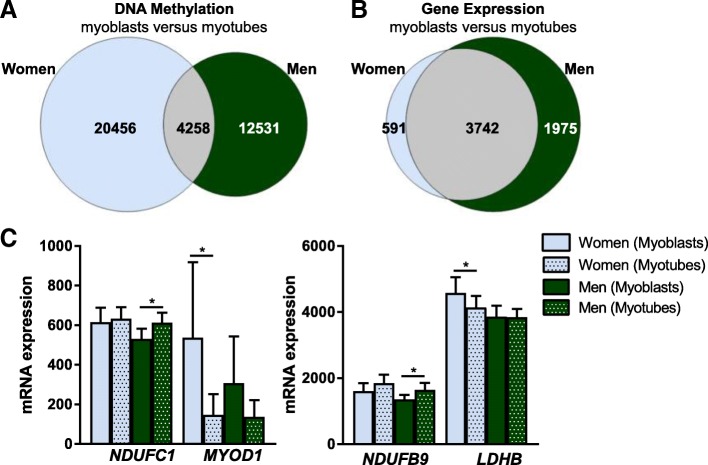
Changes in DNA methylation and gene expression during myogenesis in women and men. **a**, **b** Overlap of number of **a** CpG sites with significantly different methylation (*q <* 0.05) and **b** unique genes with significantly different gene expression (*q <* 0.05) in myoblasts versus myotubes from women (*N* = 11) and men (*N* = 13). **c** mRNA expression of selected genes with expression difference in myoblasts versus myotubes from either women or men. In women, *p* = 0.58 and *q* = 0.69 for *NDUFC1*, *p* = 0.07 and *q* = 0.14 for *NDUFB9*. In men, *p* = 0.08 and *q* = 0.14 for *MYOD1*, *p* = 0.89 and *q =* 0.92 for *LDHB*. *q < 0.05. Data are presented as mean ± SD

We have previously shown that 3.7 times more DNA methylation changes occur during differentiation of myoblasts from obese compared with non-obese subjects [12]. We therefore studied whether sex also influences changes in DNA methylation and gene expression during myogenesis. Here, we analysed methylation and expression in myoblasts versus myotubes from women and men separately and compared the results (Fig. 6a, b and Additional file 3).

In women, methylation status was altered in 24,723 CpG sites in the transition from myoblast to myotube (*q <* 0.05) (Fig. 6a). In men, methylation was altered in 16,789 sites in the same transition. Only 4258 of these sites were the same between women and men. Subsequently, most methylation changes seen during differentiation seem to be sex-specific. This was not driven by changes on the X-chromosome where relatively few changes occurred (212 in women and 262 in men, with 17 overlapping CpG sites). Interestingly, CpG sites that changed DNA methylation in women were enriched in gene bodies and open sea, while significant CpG sites in men were enriched close to TSS and in CpG islands (Additional file 4).

No CpG sites with significant difference in methylation were found on the Y chromosome in a separate analysis of DNA methylation in myoblasts versus myotubes from men (Additional file 9).

4333 unique genes in women and 5717 in men were differentially expressed between myoblasts and myotubes (*q <* 0.05) (Fig. 6b and Additional file 6). Expected expression changes of myogenic regulatory factors, muscle-specific genes and cell cycle genes during differentiation were seen for both women and men (Additional file 10) [12]. These data confirm that the myoblasts exit the cell cycle and differentiate into functional myotubes.

3742 genes with differential expression in myoblasts versus myotubes (*q <* 0.05) overlapped between women and men (Fig. 6b and Additional file 6). In line with the analysis in myoblasts and myotubes where we found differences in metabolic pathways between women and men, some genes involved in energy metabolism changed expression in either men or women during differentiation. For example, *LDHB* expression decreased in myoblasts versus myotubes from only women, while *NDUFB9* and *NDUFC1* expression increased in myoblasts versus myotubes from only men (Fig. 6c). Additionally, the expression of one myogenic regulatory factor, *MYOD1*, was only downregulated significantly in women (Fig. 6c).

Of note, three times more unique genes on the X-chromosome (68 versus 22) changed expression in men compared to women during myogenesis (Additional file 6).

Moreover, two genes on the Y-chromosome, *KDM5D* and *EIF1AY*, showed significant difference (*q <* 0.05) in gene expression between myoblasts and myotubes. Both had higher expression in myotubes (Additional file 9).

### Sex-specific DNA methylation and gene expression in human skeletal muscle

**Fig. 7:**
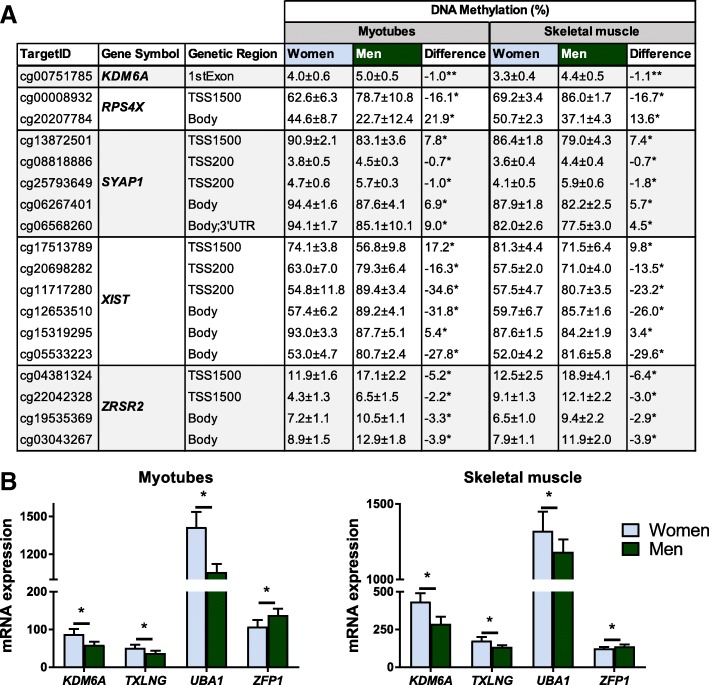
Differences in expression and methylation between women and men in and myotubes and skeletal muscle. **a** Table showing DNA methylation of significant CpG sites annotated to selected genes with differential DNA methylation and **b** bar graphs showing mRNA expression of genes with differential expression, between women and men in both myotubes and skeletal muscle. (*N* = 7–13), *q < 0.05. Data are presented as mean ± SD

Finally, we analysed if sex-specific differences in DNA methylation in myotubes can also be found in skeletal muscle tissue. Here, we used skeletal muscle from the non-diabetic individuals in a previously described cohort [28]. Methylation data were available from muscle of 7 women and 10 men. More than 50% of the differentially methylated CpG sites observed in myotubes (6539 out of 11,056) could be confirmed in muscle (*q <* 0.05) (Additional file 11). These included for example CpG sites annotated to *KDM6A*, *RPS4X*, *SYAP1, XIST* and *ZRSR2* (Fig. 7a). A great majority of the overlapping CpG sites between myotubes and skeletal muscle showed higher methylation in women, and most were found on the X-chromosome.

Expression data in muscle from the same cohort as the methylation data were available (7 women and 12 men) for 22 of the genes with differential gene expression between women and men in myotubes (Additional file 11). Four of these (*KDM6A, TXLNG*, *UBA1* and *ZFP1*) were also differentially expressed (*q <* 0.05) between women and men in whole muscle (Fig. 7b). *ZFP1* is the only autosomal gene among these (chromosome 16), and the only one with higher expression in men compared to women.

## Discussion

This study casts light on epigenetic sex differences present in human myoblasts and myotubes. Previous research has shown that sex differences are present from whole body metabolism, to tissues and on a cellular and genetic level [1–3, 13]. It is therefore important to bear in mind that sex might significantly influence the results of biomedical research. However, this factor is still often overlooked in different studies and in study designs.

Here, we found evidence that the sex origin of human myoblasts and myotubes influences their epigenome and transcriptome, which in turn can influence their differentiation and metabolism [12, 35, 39]. DNA methylation and gene expression differences between women and men that persist after myoblast differentiation into myotubes were found especially on the X-chromosome, but also on autosomal chromosomes. In addition, sex differences in DNA methylation emerged during differentiation at thousands of CpG sites, and were replicated in skeletal muscle tissue from another cohort.

Our study design, where cells from women and men were cultured under the same conditions, made it possible to study sex differences without acute effects from external factors, such as sex hormones, which might influence the results considerably [40–42]. Thus, the differences observed here are due to strictly genetic factors (XX or XY) or demonstrate an epigenetic memory from the in vivo environment. Indeed, obesity reprograms the DNA methylome of muscle stem cells [12], and elements such as sex hormones, exercise, diet and inflammatory components induce a long-term memory in the skeletal muscle [43–46]. Individuals in this study had an average BMI above normal weight (BMI > 25), which is also seen at a population level in many European countries [47]. Nevertheless, we adjusted for BMI in the statistical analyses, and PC analysis plots of methylation data did not cluster samples based on BMI or obesity. Methylation of only two CpG sites (cg02359186 and cg05801817) and expression of just one gene (*MAMSTR*) in myoblasts were associated with both sex and BMI. None of the sites/genes in myotubes significant for sex were also significantly associated with BMI. In addition, there were no difference in average BMI between women and men, and the number of obese individuals was the same in both groups. However, blood pressure and fat distribution differ between women and men in our study as expected [48–52]. These traits may affect whole body homeostasis and impact on the muscle stem cells [6]. We did not see a significant difference in VO_2_ max, an estimate of fitness levels, between the sexes after adjusting for weight. On the other hand, this has been observed previously and may require larger cohorts for detection [48, 53]. It is likely that a combination of genetic and environmental factors contributes to intrinsic sex differences in muscle stem cells. Another study has shown that knockout of the androgen receptor affects skeletal muscle of female and male mice differently [54]. Furthermore, better engraftment of muscle stem cells from female mice seems not to be caused by hormones or the immune response in the host, but innate factors in the stem cells [7]. This emphasises the importance of taking sex into account in biomedical research, as it can be of further relevance for future medicine related to muscular diseases and stem cell transplantation.

DNA methylation is known to differ between women and men, in particular due to the X-chromosome inactivation in women [13, 17, 29]. As expected, expression of *XIST*, a master regulator of X-chromosome inactivation, was significantly higher in both myoblasts and myotubes from women compared to men [13, 30]. Several CpG sites annotated to *XIST* also exhibited significant sex differences in methylation. This gene could serve as a positive control in studies regarding sex differences in gene expression and DNA methylation. Indeed, this gene has previously been suggested to serve as an expression marker, independent of tissue, for sex together with four Y-chromosome genes [55].

Epigenetic remodelling is important for cell specification and differentiation of muscle stem cells [12, 35, 39]. DNA methylation and histone modifications are known to cooperate during this process [56, 57]. We found lower methylation in *KDM6A* together with higher expression in myotubes and skeletal muscle from women versus men, in agreement with studies in other tissues [13, 17]. KDM6A is of great importance for removing repressive histone marks at muscle-specific genes during myogenesis [35]. *SIRT1*, a histone deacetylase, was also differentially methylated in myoblasts from women versus men and the expression was higher in men. A metabolic switch from fatty acid oxidation towards glycolysis during muscle stem cell activation leads to reduced NAD+ levels, which lowers SIRT1 activity [39]. This in turn leads to higher levels of H4K16 acetylation and expression of myogenic genes. Together, these indicate further differences in epigenetic regulation between women and men in myoblasts and myotubes. In future studies, it would be of interest to study whether histone modifications also differ between sexes in these cells, especially since other epigenetic enzymes changed expression in either men or women during differentiation.

The activity of epigenetic enzymes is closely linked to the metabolic state of the cell as indicated for SIRT1, and their substrate/products are mainly metabolic products [58]. GSEA showed higher expression of genes in oxidative phosphorylation in both myoblasts and myotubes from women. Oxidative metabolism enhances myoblast differentiation and muscle regeneration compared to glycolytic metabolism. In myotubes, high expression of genes in this pathway can indicate a better metabolic efficiency [39, 59, 60]. In line with this, expression of genes encoding parts of the respiratory chain (*ATP6V1B1* and *COQ3*) and genes important for pyruvate generation (*LDHB*) were higher in myoblasts from women. Women also showed higher expression of genes in pathways related to metabolism of fatty acids and amino acids in myoblasts. These pathways provide metabolites to the oxidative metabolic pathways and are expected to be upregulated during differentiation [12]. Moreover, men had higher expression of TGF-beta signalling pathway in myoblasts. TGF-beta signalling promotes myoblast proliferation and inhibits muscle differentiation [61]. Treatment of human myoblasts with TGF-β1 reduces electron transport chain capacity and complex IV abundance [62]. It is tempting to speculate that metabolic regulation during myogenesis may be sex-specific, influence epigenetic mechanisms and thereby also the differentiation itself. In support of this hypothesis, we found differential DNA methylation and expression of, e.g. *MAMSTR* and *CTF1* between women and men. *MAMSTR* showed higher expression in myoblasts from women while *CTF1* showed higher expression in men. Mamstr acts as transcriptional regulator of *MyoD* in mice [63] and *MYOD1* is a key myogenic regulatory factor that was differentially regulated during differentiation in women and men. Downregulation of *MYOD1* in only women can be an indication of shorter differentiation processes in women. However, *MYOD1* is normally not completely repressed in myotubes and has also been suggested to influence, e.g. fibre-type switch [64]. *CTF1* encodes a cytokine, CT-1, which binds to the leukaemia inhibitory factor receptor (LIFR), necessary for myoblast proliferation [65]. CT-1 also plays a role in lipid homeostasis and improves glucose uptake [66]. Overall, these data support the notion that myoblasts from male mice proliferate better, while differentiation is enhanced in females [7].

Another cellular process, autophagy, is closely related to metabolism as well as crucial for myogenesis and maintenance of muscle homeostasis [33, 67]. However, the autophagic system needs to be fine-tuned. In the present study, we found that *LAMP2*, encoding a key regulator of lysosomal function and autophagy [68], had higher expression in men compared to women in the myoblasts. This was accompanied by lower methylation levels in the proximal promoter region in men*.* With the luciferase assay, we demonstrated that higher DNA methylation in the *LAMP2* promoter directly reduces expression of the reporter gene. This implies that the methylation differences we found between women and men drive the difference observed in expression. Higher expression of *LAMP2* in myoblasts from men can be an indicator of altered autophagy compared to women, which in turn might affect differentiation and myotube function [32, 33]. There is a clear evidence of autophagic vacuoles accumulating with LAMP-2 deficiency in muscle cells [69]. Thus, DNA methylation of the *LAMP2* promoter can potentially be a way to help regulate autophagy. Of note, *LAMP2* expression in monocytes from hypogonadal men is affected by testosterone replacement therapy [70].

Increased DNA methylation in the *RPS4X* promoter also affected transcriptional activity of the reporter gene directly. *RPS4X* escapes X-chromosome inactivation and higher expression levels in women are therefore expected [34]. Indeed, we found higher *RPS4X* expression in both myoblasts and myotubes from women, together with lower methylation levels in the promoter. Thus, genes on the X-chromosome that escape inactivation in women can have lower DNA methylation than in men, and this can directly affect gene expression. This pattern with regions of lower DNA methylation on the X-chromosome in women was also seen in the global analysis of DNA methylation.

One limitation in our study is the lack of adjustment for different numbers of X-chromosomes in women and men. However, we still believe that these data are of interest since it shows differences in DNA methylation between myoblasts and myotubes from women and men, respectively. Furthermore, we find regions with higher methylation in men on the X-chromosome, despite differences in the number of this chromosome, and several of these have been described in other metabolically important tissues before [13, 17]. DNA methylation is generally tissue-specific [10]. Differences between women and men in multiple tissues therefore suggest a common regulatory mechanism and point to an importance. For example, interplays of sex chromosomes with both autosomes and mitochondria have been demonstrated and may explain sex-specific phenotypes and risk of disease [71, 72].

## Conclusions

We have shown that sex differences exist at the DNA methylome and transcriptome level in both myoblasts and myotubes, and these differences are likely to contribute to the phenotypical differences in myocytes and muscle tissue that exist between women and men.

## Additional files

Additional file 1: QC metrics and Pearson correlation of gene expression data in human myoblasts and myotubes from 11 women and 13 men. A-B Cumulative density plotted against log 2 intensity of expression data (A) before and (B) after normalisation. C-D Quantile normalisation of signal intensities (C) before and (D) after normalisation. E-F Person correlation between expression data of all samples in E) myoblasts and F) myotubes. (PDF 301 kb)

Additional file 2: Clustering of samples based on PC analyses of methylation data in human myoblasts and myotubes from 13 women and 13 men. x-axis shows PC2 and y-axis shows PC1. (PDF 48 kb)

Additional file 3: DNA methylation data from the Infinium HumanMethylation450K BeadChip (Illumina) from 13 women and 13 men. Different Excel sheets correspond to different comparison, i.e. myoblasts (women vs. men), myotubes (women vs. men), women (myoblasts vs. myotubes) and men (myoblasts vs. myotubes). (XLSX 1020 kb)

Additional file 4: Distribution and frequency of CpG sites with significant difference in DNA methylation between women and men. A-D Frequencies of CpG sites that exhibit significant (*q <* 0.05) DNA methylation differences in women versus men in myoblasts and myotubes on the X-chromosome and the autosomal chromosomes in functional gene regions and CpG island regions. E-F) Frequencies of CpG sites that exhibit significant (*q <* 0.05) DNA methylation differences in myoblasts versus myotubes in women and men in functional gene regions and CpG island regions. Frequencies are compared to all analysed CpG sites using chi-square tests. *q < 0.05 in comparison to all analysed, ¤*q <* 0.05 for women versus men; TSS200/TSS1500: proximal promoter at 0–200 bp and 200–1500 bp, respectively, upstream of the transcription start site (TSS); UTR: untranslated region; CpG island: 200 bp (or more) stretch of DNA with GC content > 50% and CpG observed/expected ratio higher than 0.6; Shore: region of 2000 bp directly flanking the CpG island upstream (northern (N)) or southern (downstream (S)); Shelf: regions of 2000 bp flanking the island shores. (PDF 48 kb)

Additional file 5: Results from GSEA of enriched KEGG pathway. Results from GSEA of KEGG pathways based on expression data for women versus men in myoblasts and myotubes, respectively. FDR *q* values were used to determine pathway enrichment (*q <* 0.05). (XLSX 13 kb)

Additional file 6: mRNA expression data from the HumanHT-12 Expression BeadChip (Illumina) in myoblasts and myotubes from 11 women and 13 men. Different Excel sheets correspond to different comparisons, i.e. myoblasts (women vs. men), myotubes (women vs. men), women (myoblasts vs. myotubes) and men (myoblasts vs. myotubes). (XLSX 2430 kb)

Additional file 7: Overlap between significant DNA methylation data and mRNA expression data in myoblasts and myotubes. DNA methylation and mRNA expression merged on annotated gene symbol for genes with significant differences in gene expression. Different Excel sheets correspond to different comparison, i.e. myoblasts (women vs men) and myotubes (women vs men), women (myoblasts vs. myotubes) and men (myoblasts vs. myotubes). (XLSX 2880 kb)

Additional file 8: Average DNA methylation in myoblasts and myotubes from 13 women and 13 men. Average DNA methylation of CpG sites annotated to functional gene regions and CpG island regions on autosomal chromosomes and the X-chromosome, respectively. Data are presented as mean ± SD. *q < 0.05. (PDF 107 kb)

Additional file 9: DNA methylation and mRNA expression data on the Y-chromosome in myoblasts versus myotubes from 13 men. Different Excel sheets correspond to DNA methylation data and mRNA expression data respectively. (XLSX 61 kb)

Additional file 10: mRNA expression in myoblasts versus myotubes from 11 women and 13 men of A myogenic regulatory factors, B myocyte enhancer factor-2 genes, C-D muscle-specific genes and E-F cell cycle genes. Data are presented as mean ± SEM. *q < 0.05. (PDF 171 kb)

Additional file 11: DNA methylation and mRNA expression data in skeletal muscle from women (*N* = 7) and men (*N* = 10–12). Different Excel sheets correspond to DNA methylation data and mRNA expression data respectively. (XLSX 1230 kb)

## Abbreviations

BMI: Body mass index
BMIQ: Beta-mixture quantile normalisation
bp: Base pair
FBS: Fetal bovine serum
FDR: False discovery rate
Gnrh: Gonadotropin-releasing hormone
GSEA: Gene set enrichment analysis
H3K27ac: Histone 3 acetylation on lysine 27
H3K4me1: Histone 3 methylation on lysine 4
H3K4me3: Histone 3 trimethylation on lysine 4
HOMA-B: Homeostasis model assessment of β-cell function
HOMA-IR: Homeostasis model assessment of insulin resistance
HSMM: Skeletal muscle myoblasts
Ile: Isoleucine
Leu: Leucine
N shelf: Northern shelf
N shore: Northern shore
PC: Principal component
PS: Penicillin/streptomycin
QC: Quality control
S shelf: Southern shelf
S shore: Southern shore
SD: Standard deviation
TGF-beta: Transforming growth factor-beta
TSS: Transcription start site
Val: Valine
