# Supplementary figures and images for: Sex influences DNA methylation and gene expression in human skeletal muscle myoblasts and myotubes
Source: Stem Cell Res Ther. 2019 Jan 15;10:26. doi: 10.1186/s13287-018-1118-4 (PMC6332625; doi:10.1186/s13287-018-1118-4)

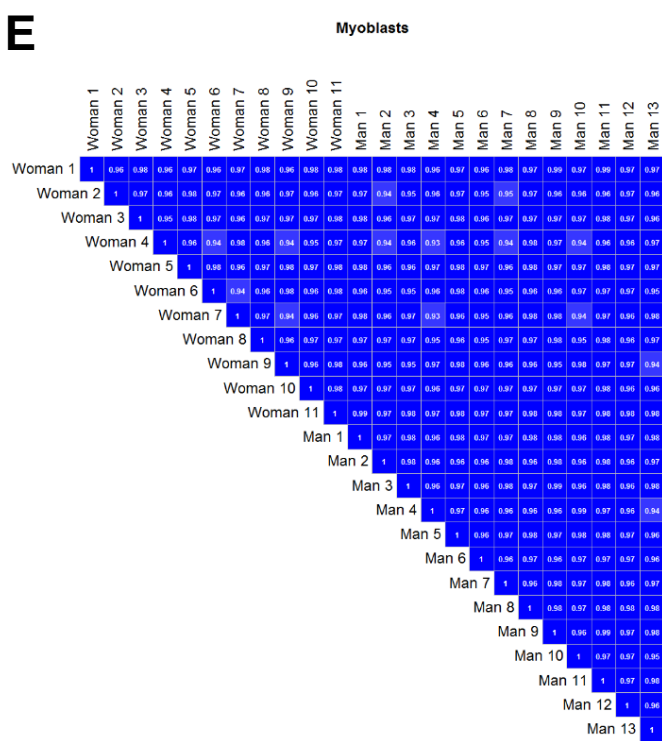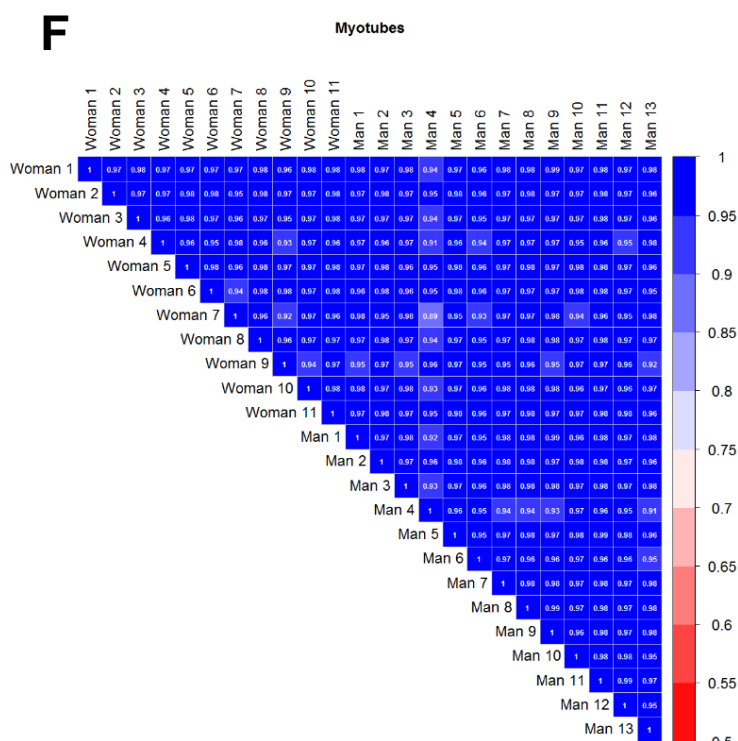

Supplement: Supplementary file 1 — QC metrics and Pearson correlation of gene expression data in human myoblasts and myotubes from 11 women and 13 men. A-B Cumulative density plotted against log 2 intensity of expression data (A) before and (B) after normalisation. C-D Quantile normalisation of signal intensities (C) before and (D) after normalisation. E-F Person correlation between expression data of all samples in E) myoblasts and F) myotubes. (PDF 301 kb) [file 13287_2018_1118_MOESM1_ESM.pdf]

Myoblasts

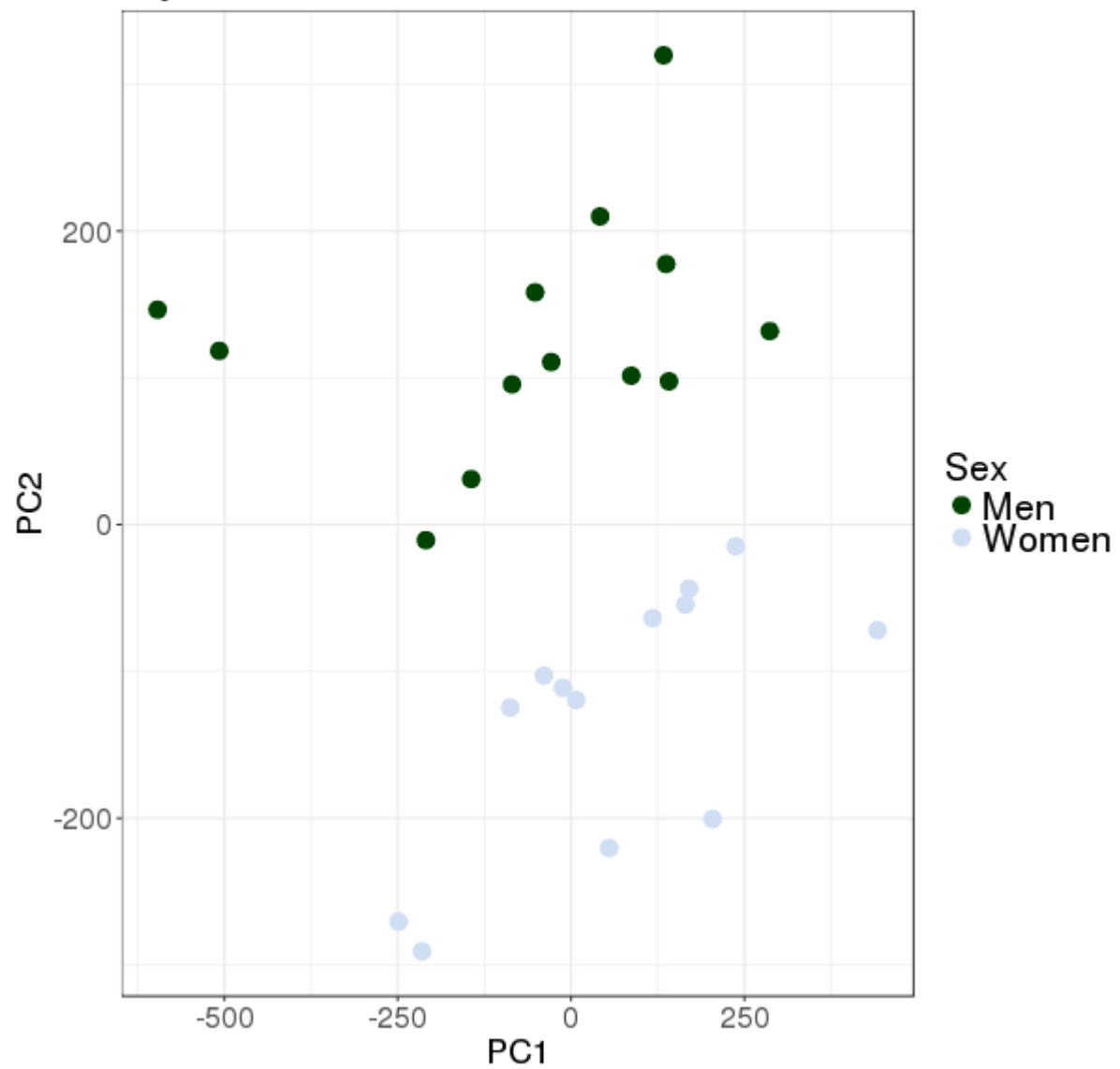

Myotubes

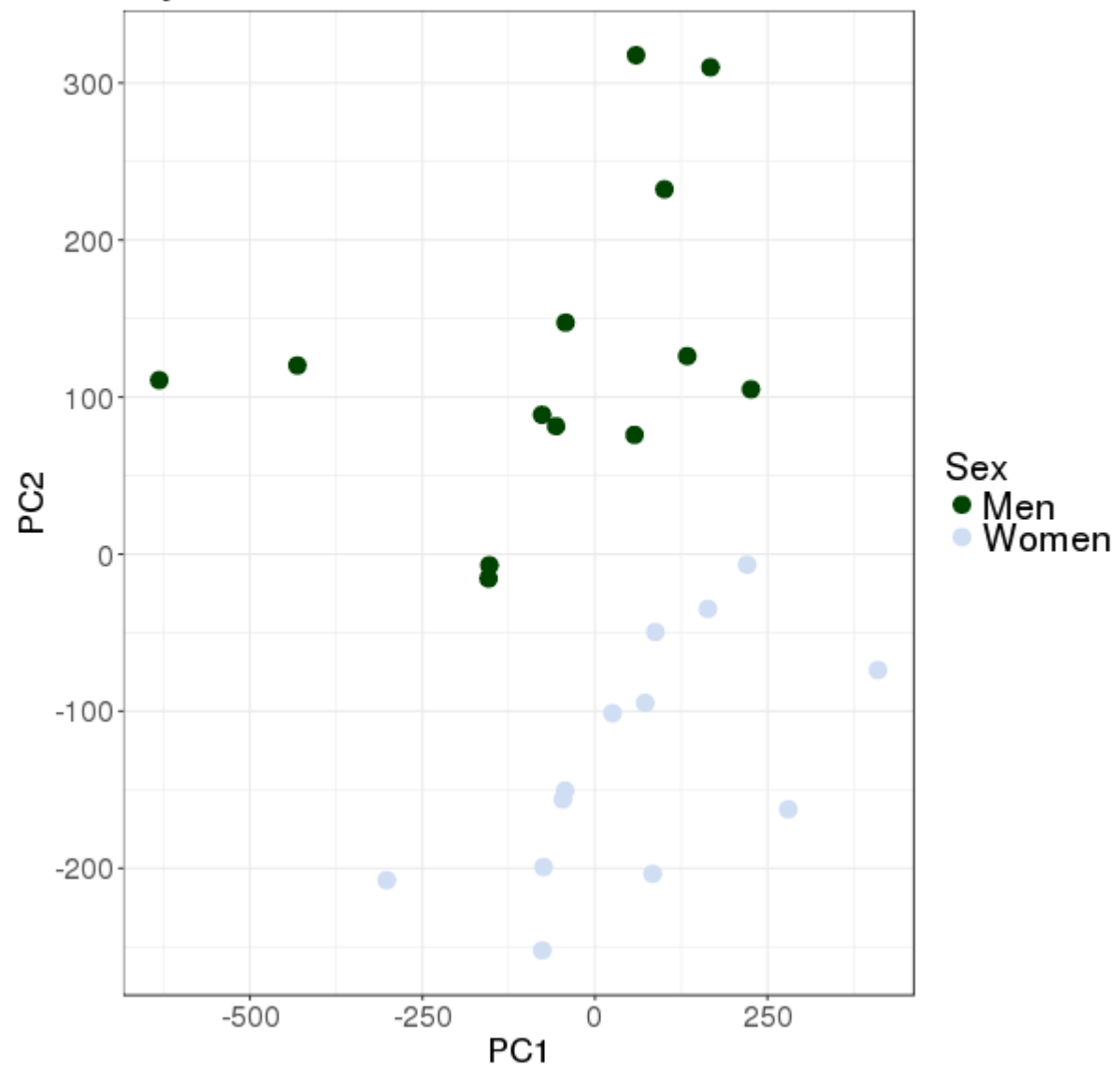

Supplement: Supplementary file 2 — Clustering of samples based on PC analyses of methylation data in human myoblasts and myotubes from 13 women and 13 men. x-axis shows PC2 and y-axis shows PC1. (PDF 48 kb) [file 13287_2018_1118_MOESM2_ESM.pdf]

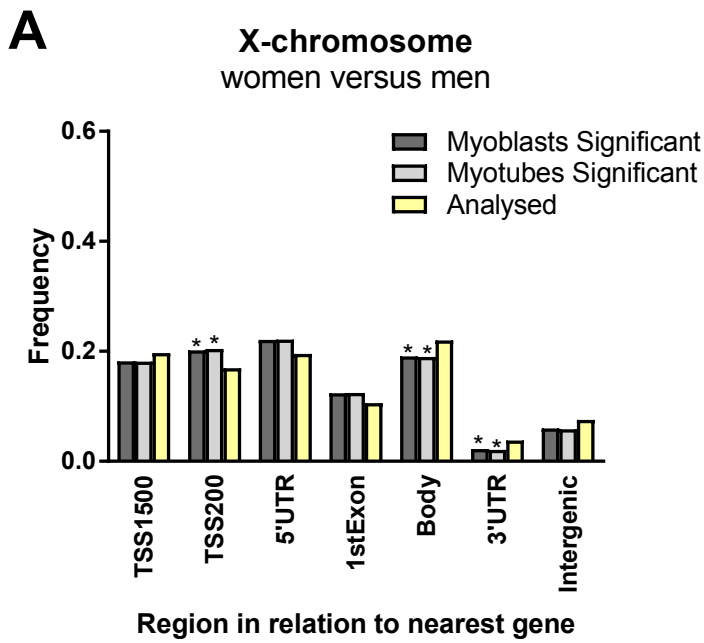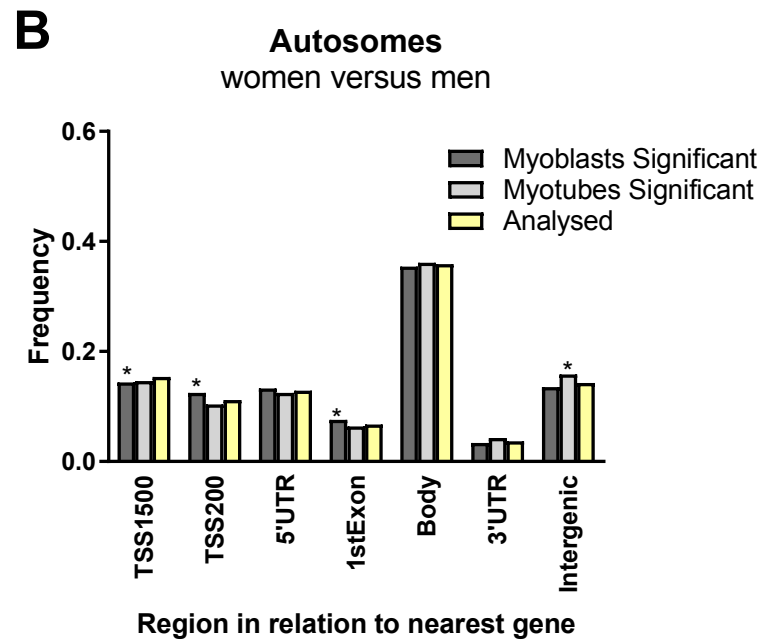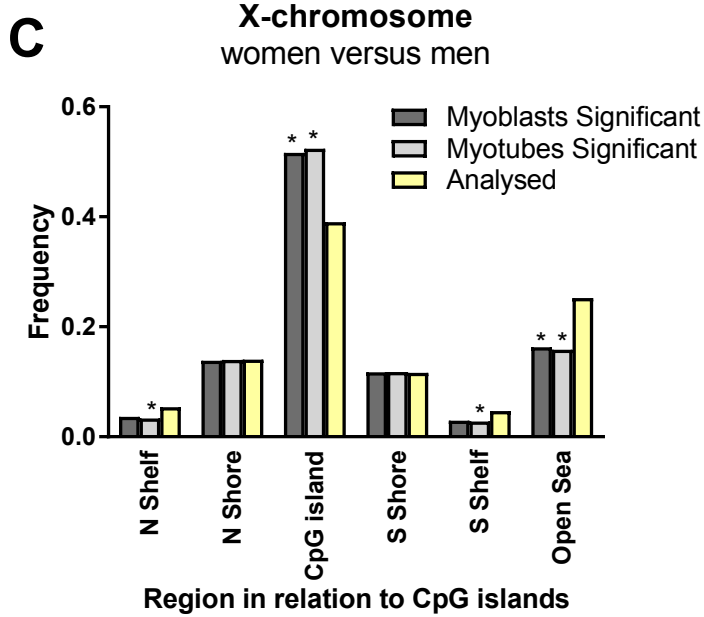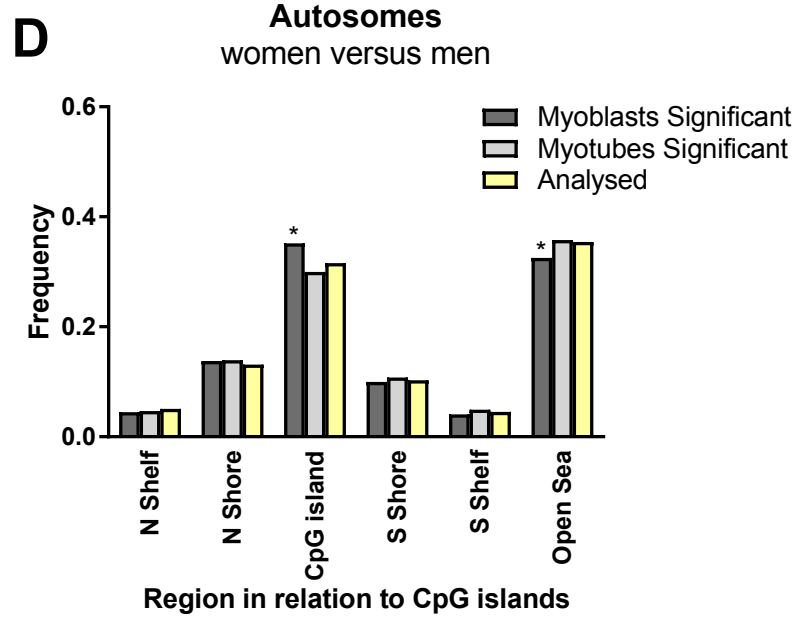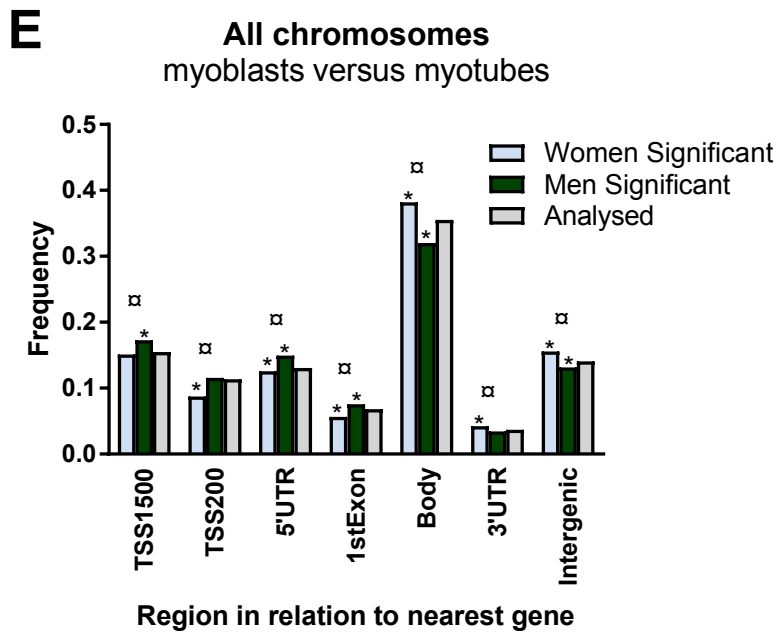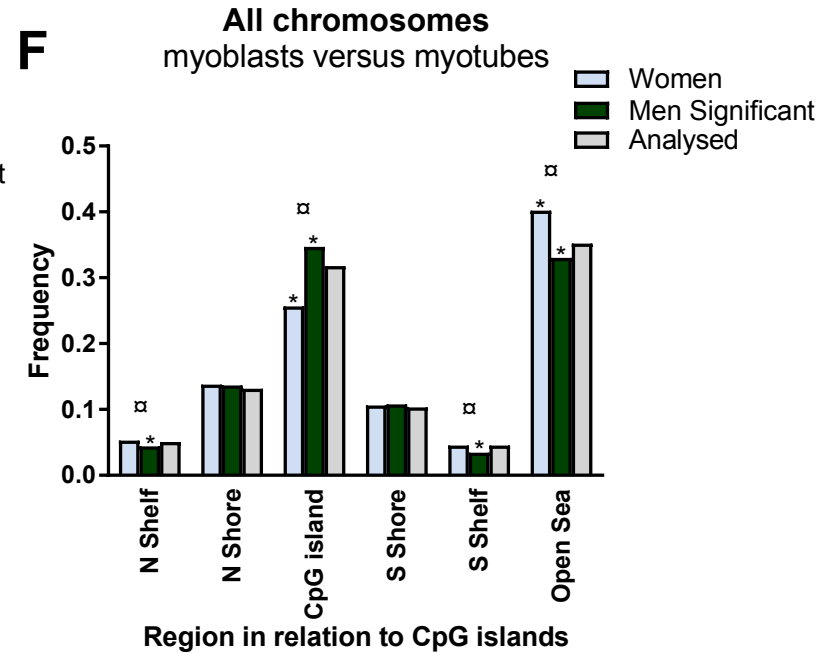

Supplement: Supplementary file 4 — Distribution and frequency of CpG sites with significant difference in DNA methylation between women and men. A-D Frequencies of CpG sites that exhibit significant (q < 0.05) DNA methylation differences in women versus men in myoblasts and myotubes on the X-chromosome and the autosomal chromosomes in functional gene regions and CpG island regions. E-F) Frequencies of CpG sites that exhibit significant (q < 0.05) DNA methylation differences in myoblasts versus myotubes in women and men in functional gene regions and CpG island regions. Frequencies are compared to all analysed CpG sites using chi-square tests. *q < 0.05 in comparison to all analysed, ¤q < 0.05 for women versus men; TSS200/TSS1500: proximal promoter at 0–200 bp and 200–1500 bp, respectively, upstream of the transcription start site (TSS); UTR: untranslated region; CpG island: 200 bp (or more) stretch of DNA with GC content > 50% and CpG observed/expected ratio higher than 0.6; Shore: region of 2000 bp directly flanking the CpG island upstream (northern (N)) or southern (downstream (S)); Shelf: regions of 2000 bp flanking the island shores. (PDF 48 kb) [file 13287_2018_1118_MOESM4_ESM.pdf]
