# Supplementary material for: Sex influences DNA methylation and gene expression in human skeletal muscle myoblasts and myotubes
Source: Stem Cell Res Ther. 2019 Jan 15;10:26. doi: 10.1186/s13287-018-1118-4 (PMC6332625; doi:10.1186/s13287-018-1118-4)
Supplement: Supplementary file 8 — Average DNA methylation in myoblasts and myotubes from 13 women and 13 men. Average DNA methylation of CpG sites annotated to functional gene regions and CpG island regions on autosomal chromosomes and the X-chromosome, respectively. Data are presented as mean ± SD. *q < 0.05. (PDF 107 kb) [file 13287_2018_1118_MOESM8_ESM.pdf]

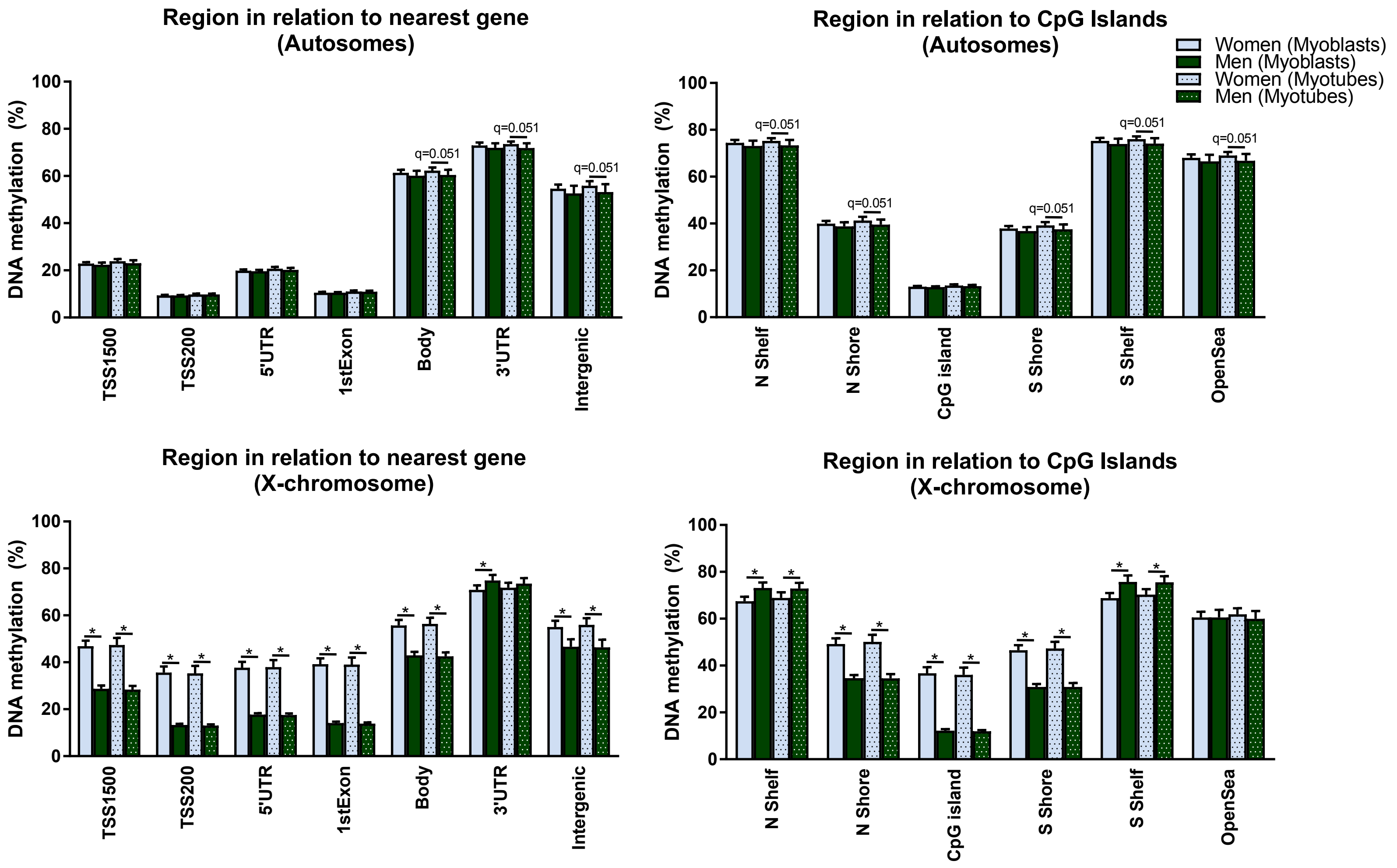
Region in relation to nearest gene
(Autosomes)
Region in relation to CpG Islands
(Autosomes)
Women (Myoblasts)
Men (Myoblasts)
Women (Myotubes)
Men (Myotubes)
DNA methylation (%)
q=0.051
TSS1500
TSS200
5'UTR
1stExon
Body
3'UTR
Intergenic
N Shelf
N Shore
CpG island
S Shore
S Shelf
OpenSea
Region in relation to nearest gene
(X-chromosome)
Region in relation to CpG Islands
(X-chromosome)
