# Supplementary material for: Sex influences DNA methylation and gene expression in human skeletal muscle myoblasts and myotubes
Source: Stem Cell Res Ther. 2019 Jan 15;10:26. doi: 10.1186/s13287-018-1118-4 (PMC6332625; doi:10.1186/s13287-018-1118-4)
Supplement: Supplementary file 10 — mRNA expression in myoblasts versus myotubes from 11 women and 13 men of A myogenic regulatory factors, B myocyte enhancer factor-2 genes, C-D muscle-specific genes and E-F cell cycle genes. Data are presented as mean ± SEM. *q < 0.05. (PDF 171 kb) [file 13287_2018_1118_MOESM10_ESM.pdf]

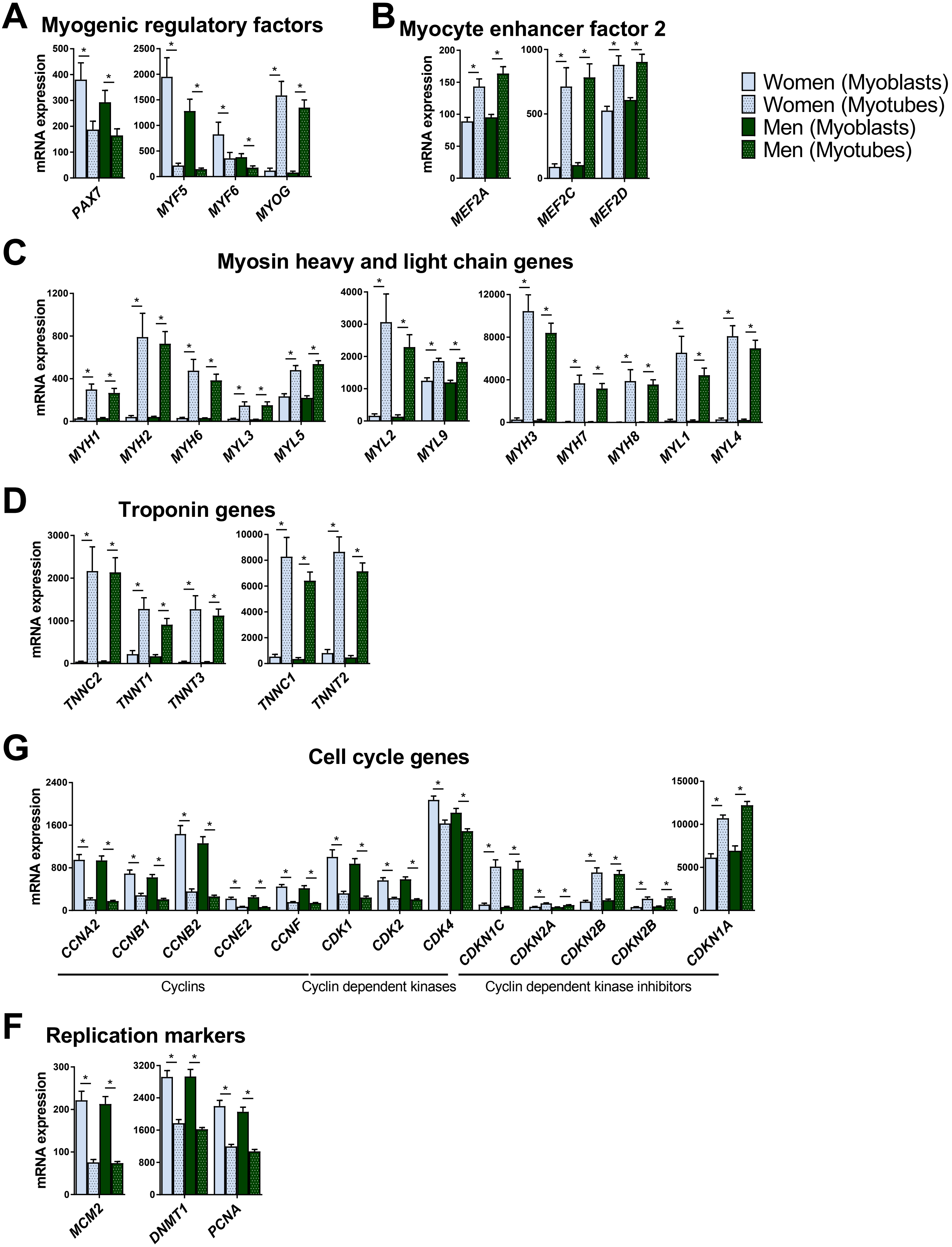
A
Myogenic regulatory factors
B
Myocyte enhancer factor 2
Women (Myoblasts)
Women (Myotubes)
Men (Myoblasts)
Men (Myotubes)
mRNA expression
PAX7
MYF5
MYF6
MYOG
MEF2A
MEF2C
MEF2D
C
Myosin heavy and light chain genes
MYH1
MYH2
MYH6
MYL3
MYL5
MYL2
MYL9
MYH3
MYH7
MYH8
MYL1
MYL4
D
Troponin genes
TNNC2
TNNT1
TNNT3
TNNC1
TNNT2
G
Cell cycle genes
CCNA2
CCNB1
CCNB2
CCNE2
CCNF
CDK1
CDK2
CDK4
CDKN1C
CDKN2A
CDKN2B
CDKN2B
CDKN1A
Cyclins
Cyclin dependent kinases
Cyclin dependent kinase inhibitors
F
Replication markers
MCM2
DNMT1
PCNA
